# Supplementary material for: Natural hybridization in heliconiine butterflies: the species boundary as a continuum
Source: BMC Evol Biol. 2007 Feb 23;7:28. doi: 10.1186/1471-2148-7-28 (PMC1821009; doi:10.1186/1471-2148-7-28)
Supplement: Additional File 1 — Hybrids between species of Heliconius and Eueides butterflies: a database. HTML file linking to database of all known wild-caught interspecific hybrid specimens in the Heliconiina, consisting of introductory text, a list of specimens, together with collection data and photographs of the specimens, and links to information about some artificial hybrids and mutants in the group. This is an edited copy of our online database of Heliconius hybrids [102]. To view database, download zip file and extract to a separate folder, then open index.html within that folder. [file 1471-2148-7-28-S1.zip › isavib04.html]

hybrid isavib04


---


Hybrid between *Eueides isabella eva* and *E. vibilia vialis*
Mexico
© S. Knapp

Return to table
of hybrids

To next hybrid
  
To previous hybrid

```
NOTES

No:                      5
Genus of species 1:      Eueides
Species 1:               isabella
Subspecies of species 1: eva
Genus of species 2:      Eueides
Species 2:               vibilia
Subspecies of species 2: vialis
Sex:                     m
Country:                 Mexico
Locality:                Oaxaca: Chiltepec
Year:                    1975
Photo no.:               isavib04
Named hybrid:
Collection:              UNAM
Collector:               A. D�az Franc�s
Author/publication:
Notes:                   F1, presumably
```

**Last updated:** 18 October 2003

---
